# Health Culture

**Published:** 1894-12

**Authors:** 


HEALTH CULTURE: A journal of Practical Hygiene. The Health Culture Co., No. 30 East Fourteenth Street, Room No. 20, New York. Terms, 50 cents a year; 15 cents a number.

This publication is a quarterly journal devoted to teaching hygiene to the laity. The second number is before us and among other things contains an article on the home treatment of fevers; an article advocating vegetarianism; the Swedish system of gymnastics; full deep breathing and its benefits, and various other articles of similar import.

